# Evoked delayed potential ablation for post-myocardial infarction ventricular tachycardia: results from a large prospective multicentre study

**DOI:** 10.1093/europace/euaf003

**Published:** 2025-02-24

**Authors:** Marta de Riva, Reinder Evertz, Peter Lukac, Lukas R C Dekker, Yuri Blaauw, Rachel M A ter Bekke, Yoshitaka Kimura, Rypko J Beukema, Alexandre Ouss, Bart A Mulder, Kevin Vernooy, Adrianus P Wijnmaalen, Katja Zeppenfeld

**Affiliations:** Department of Cardiology, Willem Einthoven Center of Arrhythmia Research and Management, Leiden University Medical Center, Postbus 9600, Leiden 2300 RC, The Netherlands; Department of Cardiology, Radboud University Medical Center, Nijmegen, The Netherlands; Department of Cardiology, Aarhus University Hospital, Aarhus, Denmark; Department of Cardiology, Catharina Hospital, Eindhoven, The Netherlands; Department of Cardiology, University Medical Center Groningen, Groningen, The Netherlands; Department of Cardiology. Maastricht University Medical Center, Cardiovascular Research Institute Maastricht, Maastricht, The Netherlands; Department of Cardiology, Willem Einthoven Center of Arrhythmia Research and Management, Leiden University Medical Center, Postbus 9600, Leiden 2300 RC, The Netherlands; Department of Cardiology, Radboud University Medical Center, Nijmegen, The Netherlands; Department of Cardiology, Catharina Hospital, Eindhoven, The Netherlands; Department of Cardiology, University Medical Center Groningen, Groningen, The Netherlands; Department of Cardiology. Maastricht University Medical Center, Cardiovascular Research Institute Maastricht, Maastricht, The Netherlands; Department of Cardiology, Willem Einthoven Center of Arrhythmia Research and Management, Leiden University Medical Center, Postbus 9600, Leiden 2300 RC, The Netherlands; Department of Cardiology, Willem Einthoven Center of Arrhythmia Research and Management, Leiden University Medical Center, Postbus 9600, Leiden 2300 RC, The Netherlands

**Keywords:** Ventricular tachycardia, Myocardial infarction, Substrate ablation, Substrate modification, Functional substrate mapping, Evoked delayed potentials

## Abstract

**Aims:**

The optimal substrate ablation approach for post-myocardial infarction (MI) ventricular tachycardia (VT) is unknown. Proposed ablation targets are prone to individual interpretation making the ablation outcome potentially operator dependent. Evoked delayed potentials (EDPs) are a well-defined target. Evoked delayed potential ablation was effective in preventing post-MI VT recurrence in a prior study. The aims of this study were to assess long-term outcomes of EDP ablation in a large multicentre cohort of post-MI patients and to compare ablation outcomes between centres with and without prior experience in EDP ablation.

**Methods and results:**

Patients with post-MI VT undergoing ablation in one centre performing EDP ablation since 2013 and five centres without prior experience in EDP ablation were prospectively included. A uniform mapping protocol including right ventricular extra-stimulation aiming to EDP identification was followed. Ablation endpoints were EDP elimination and VT non-inducibility. Patients were followed for VT recurrence, mortality, heart transplant, and left ventricular assist device implantation. In total, 130 patients were included. The protocol was successfully performed in 99%, and in 94%, EDPs were identified and ablated. In total, 78% of patients were rendered non-inducible. Ventricular tachycardia-free survival was 78% [95% confidence interval (CI) 71–85] and 71% (95% CI 63–80) at 6 and 12 months, respectively. No difference in VT-free survival was observed among centres with and without prior experience in EPD ablation.

**Conclusion:**

In a large multicentre prospective cohort of patients with post-MI VT, EDP ablation resulted in good long-term outcomes. Importantly, VT recurrence rates did not differ among centres with and without prior experience in EDP ablation, indicating that this approach can be easily reproduced by operators previously not familiar with the technique.

## Graphical Abstract

**Graphical Abstract:**
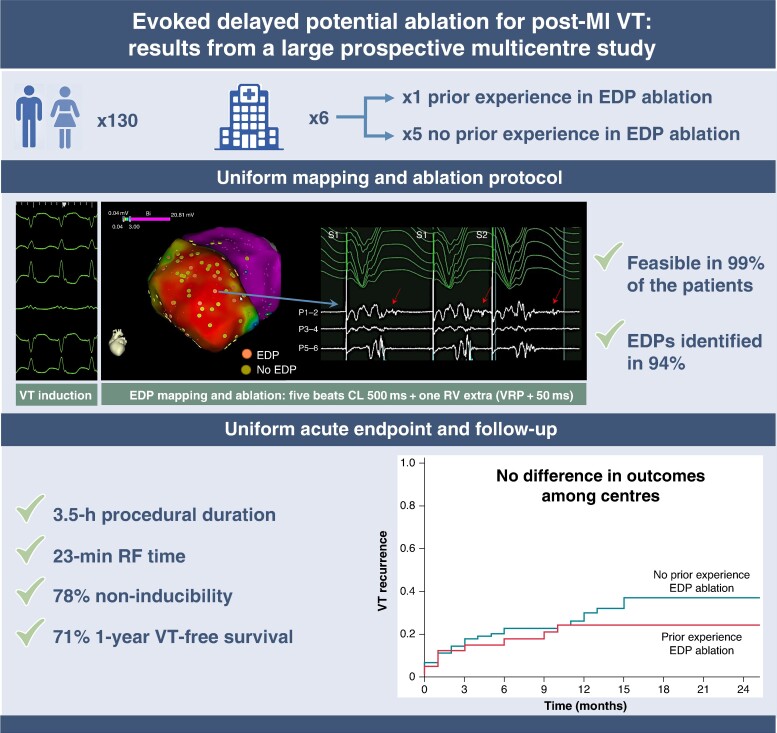

**What’s new?**

- In a multicentre prospective observational cohort of patients with post-myocardial infarction ventricular tachycardia (VT), evoked delayed potential ablation was associated with low VT recurrence rates.
- There were no differences in outcomes among patients undergoing the procedure in a centre with large prior experience with EDP ablation and patients treated in centres without prior experience with the technique.
- Procedural and ablation times using this substrate modification approach were short (a median of 210 and 23 min, respectively).

## Introduction

Substrate-based ablation for the treatment of scar-related ventricular tachycardia (VT) relies on the identification and elimination of areas of scar that may serve as potential sources for VT during stable (sinus) rhythm. In patients with prior myocardial infarction (MI), substrate ablation has been shown to be superior to ablation approaches targeting only clinical and haemodynamically tolerated VTs and has therefore become the standard ablation strategy for post-MI patients in many electrophysiology laboratories.^1–3^

However, the optimal substrate modification approach for post-MI VT is currently unknown. Various techniques have been proposed, which generally aim to identify and target low-voltage electrograms (EGMs) consistent with slow conduction and/or poorly coupled EGMs considered to be involved in VT initiation and maintenance.^4–7^ However, the evidence on the acute and long-term efficacy of these ablation approaches comes from studies conducted in high-volume centres, the majority of which are retrospective, often encompassing mixed populations of patients with ischaemic and non-ischaemic cardiomyopathies.^5,7–9^ Additionally, proposed ablation targets often have variable and/or broader definitions, requiring individual interpretation and making procedural outcomes potentially dependent on operator proficiency.^5,6^

In a single-centre retrospective study, a functional substrate mapping approach aiming for identification and ablation of evoked delayed potentials (EDPs) was highly effective in preventing post-MI VT recurrence.^10^ Evoked delayed potentials were specifically defined as low-voltage near-field potentials that delayed more than 10 ms or blocked in response to a short-coupled right ventricular (RV) extra-stimulus, indicating the presence of electrophysiological properties (slow conduction and block) necessary for reentrant VT.

We hypothesized that substrate ablation for post-MI VT based on EDP identification as a well-defined and measurable target can be easily reproduced with comparable outcomes in centres without prior experience with this ablation strategy. The objectives of this study were two-fold:

- To assess the acute and long-term outcome of EDP ablation in a large prospective cohort of patients with post-MI VT.
- To compare long-term outcomes of EDP ablation among centres with and without prior experience with this ablation approach.

## Methods

### Study design

This is a prospective, international, multicentre cohort study. Patients with post-infarct VTs were enrolled from six centres to evaluate the outcome after ablation of patients with post-infarct VT following a pre-specified substrate modification approach based on EDP ablation. Outcomes were compared between centres with and without prior experience with this ablation approach. Patients were included in the study and analysed according to an intention to treat principle.

### Study patients

Consecutive patients accepted for ablation of symptomatic sustained monomorphic VT after MI were included. The diagnosis of MI was based on the clinical history and the presence of wall motion abnormalities, non-reversible perfusion defects, and/or subendocardial or transmural late gadolinium enhancement areas in the perfusion territory of a significantly stenotic coronary artery (>75%). Patients with scar-related VT due to non-ischaemic aetiologies and carriers of left ventricular assist devices (LVADs) were excluded. Patients who underwent an electrophysiological study with left ventricular (LV) mapping, also if ablation was not performed, were included in the analysis. All patients provided informed consent for the procedure and for inclusion in a prospective registry. The study was approved by the Medical Ethics Committee of Leiden The Hague Delft (N.19069) and subsequently by the ethics committee of each participating centre.

Medical records were reviewed for demographic and clinical patient characteristics including past ischaemic events, prior revascularization strategies, treatment for heart failure and arrhythmias, including anti-arrhythmic drugs (AADs), and prior ablation procedures. Data on arrhythmia presentation, mapping and ablation, acute ablation endpoints, and complications as well as follow-up data regarding VT recurrence, repeat ablation procedures, mortality, cause of death, and use of AADs were collected. All data were anonymized and entered into a secured online database.

### Study centres

One of the six enrolling centres is a high-volume referral centre for VT ablation performing EDP-based substrate ablation in post-MI patients since 2013.^10^ The other five centres did not have prior experience with this ablation approach and started to perform EDP substrate modification when the study was started in 2019 after a short training.

### Pre-procedural evaluation and management

Anti-arrhythmic drugs with the exception of amiodarone were stopped five half-lives before the procedure. An echocardiogram was performed to evaluate LV function, assess the presence of significant valvular disease, and exclude LV thrombus. Pre-procedural imaging with late gadolinium-enhanced contrast magnetic resonance (LGE-CMR) to evaluate scar location, extension, and transmurality or with cardiac computed tomography (CCT) to assess areas of wall thinning was encouraged.

### Electrophysiological study, mapping, and ablation

**Figure 1:**
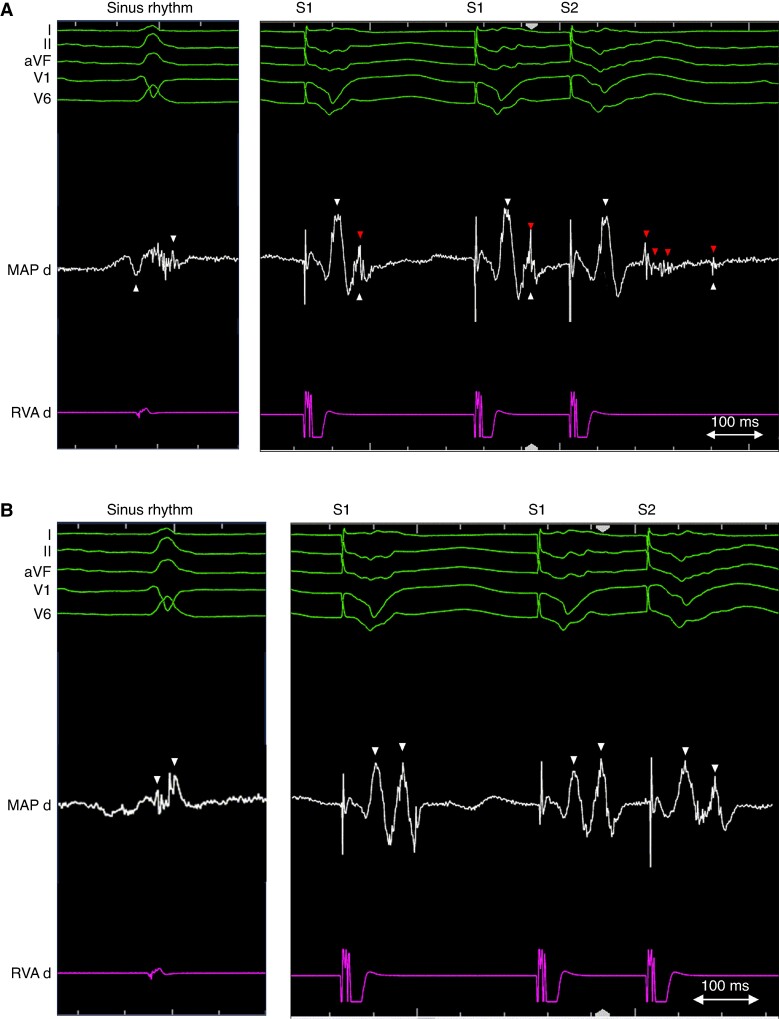

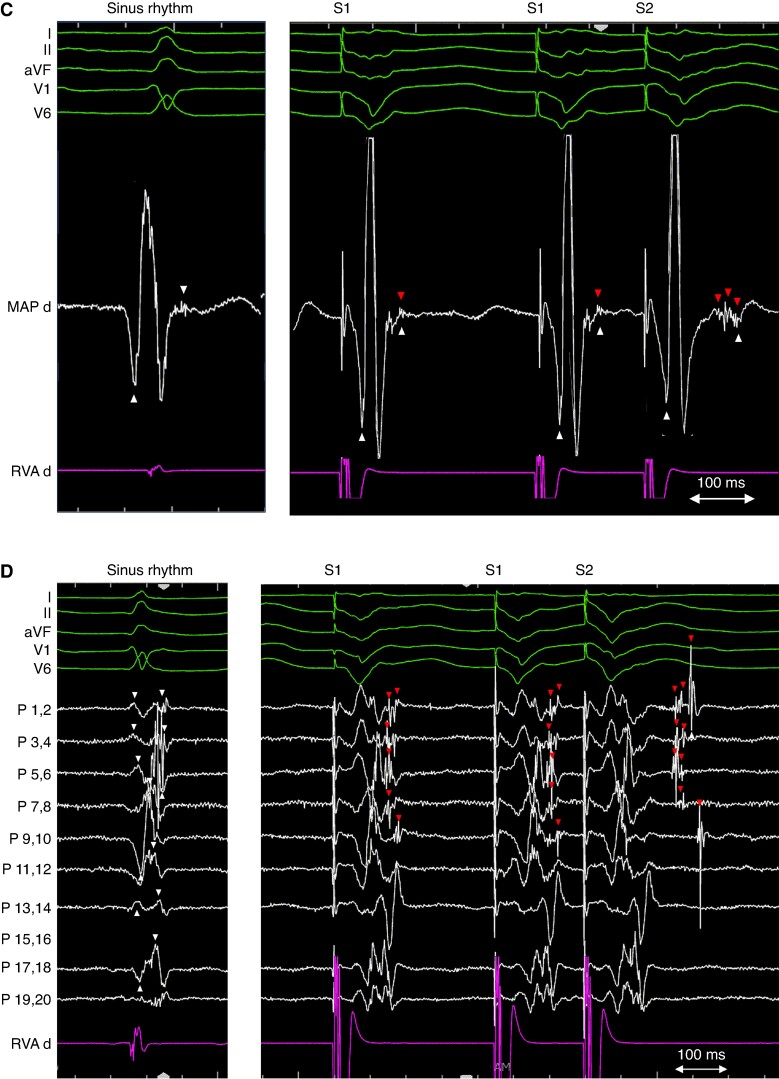
Examples of left ventricular EGM responses to RV extra-stimulation. (*A*) EDP site: during sinus rhythm (SR), a low-voltage EGM with fragmentation is recorded (left panel). During S1 (centre), two EGM components become visible: a low-frequency potential compatible with far-field and a high-frequency component compatible with near-field activity. During S2 (right), the near-field potential delays and splits into multiple components. (*B*) No EDP site: During SR, a low-voltage, fragmented EGM is recorded. During S1, two EGM components separate but do not delay further with the application of the extra-stimulus (S2). (*C*) EDP site: during SR, two EGM components can be distinguished: a high-voltage far-field EGM and a near-field EGM at the end of the QRS complex. During S1, no further delay of the near-field is appreciated, but it delays significantly during S2. (*D*) Bipolar EGMs recorded during SR and during RV pacing (S1 and S2) with a multipolar penta-spline multipolar catheter. During SR, fragmentation is observed in practically all bipoles. During S1, local near-field potentials become more evident in bipoles from 1–2 to 9–10, whereas bipoles from 13–14 to 19–20 show low-frequency potentials. During S2, near-field components delay and split further (EDPs) in consecutive bipoles from 1–2 to 9–10 but no delay is observed in bipoles from 13–14 to 19–20. The white triangles indicate the earliest and latest sharp peaks of the bipolar EGMs, and redt triangles indicate fragmented and delayed near-field potentials. EDP, evoked delayed potential; EGM, electrogram; RV, right ventricle.

**Figure 2:**
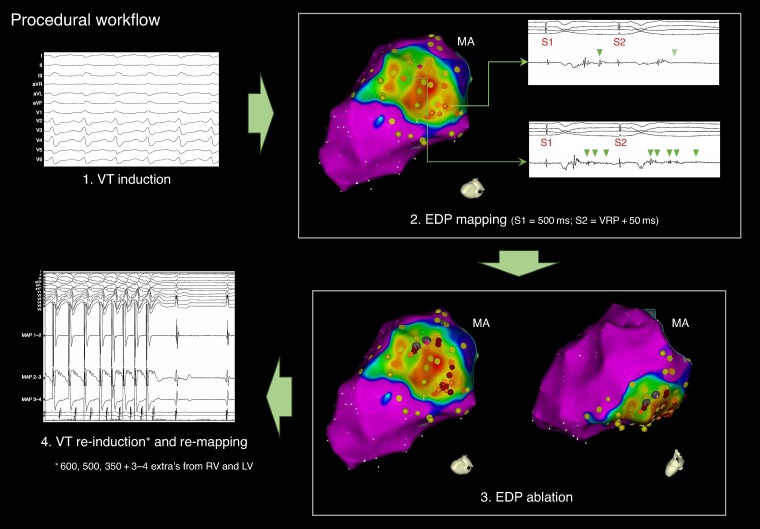
Procedural workflow. EDP, evoked delayed potential; LV, left ventricle; RV, right ventricle; VRP, ventricular refractory period; VT, ventricular tachycardia.

Procedures were performed under conscious sedation, deep sedation, or general anaesthesia according to patient characteristics, physician preference, and local availability. Use of haemodynamic support and image (LGE-CMR/CCT) integration was left at operator’s discretion. Before mapping and ablation, programmed electrical stimulation (PES) was performed in an attempt to induce VT [four drive cycle lengths (CLs) of 600, 500, 400, and 350 ms and 1–4 ventricular extra-stimuli down to ventricular refractory period (VRP) from at least one RV site and one LV site]. Positive endpoint of stimulation was the induction of sustained monomorphic VT lasting >30 s or requiring termination because of haemodynamic instability. An induced VT was regarded clinical if the 12-lead electrocardiogram (ECG) matched that of a spontaneous VT and presumptive clinical when the CL was +/−30 ms and the far-field EGM morphology matched that of an implantable cardioverter–defibrillator (ICD)-recorded VT. Any other induced VT was considered non-clinical. Fast VT was defined as a VT with a CL ≤ VRP +30 ms.^11^ Electroanatomical LV endocardial mapping was performed in all patients via a retrograde approach, a transseptal approach, or a combination of both depending on operator’s preference. Additional endocardial RV mapping and epicardial mapping were performed when deemed appropriate. Use of multipolar catheters for high-density mapping was encouraged. Bipolar voltage (BV) maps were created and low-voltage areas were typically defined as <1.5 mV when multipolar catheters were used and BV <3 mV (for patients with preserved or mildly reduced ejection fraction) or BV <2.1 mV (for patients with reduced ejection fraction) when single-tip catheters were used for mapping.^12^ All EGMs located in infarcted areas based on the clinical history and pre-procedural imaging data, irrespective of their voltage or morphology, were systematically analysed during sinus rhythm, RV pacing at a fixed rate of 500 ms, and during the application of a single RV extra-stimulus with a coupling interval of 50 ms above the VRP. Sites exhibiting low-amplitude near-field potentials which delayed >10 ms or blocked in response to the short-coupled RV extra-stimulus were categorized as EDPs and annotated on the map. Examples of EDPs are given in *Figure 1*. Substrate modification aiming at EDP elimination was performed. If during catheter manipulation or during the performance of the pacing protocol, a tolerated sustained VT was repeatedly induced, activation and entrainment mapping was performed with the aim of identifying the VT isthmus and terminating the VT by ablation. Radiofrequency (RF) energy was delivered with 40–50 W, a temperature limit of 43–45°C, and a flow rate of 15–30 mL/min. Use of irrigated contact forced ablation catheters was preferred aiming for a contact force >9 g. The procedural workflow is summarized in *Figure 2*. Representative examples of two patients with inferior MI undergoing EDP ablation are given in Supplementary material online, *Figure S1*.

### Ablation endpoints

After the last RF application, a re-map including RV extra-stimulation to verify EDP elimination was performed and the entire induction protocol was repeated. Fast VTs with a CL close to VRP were considered clinically not relevant and not targeted.^11^ If any other sustained monomorphic VT remained inducible, additional mapping and ablation was performed until no further substrate could be identified.

### Follow-up

Patients were followed 3 months after ablation and every 6 months afterwards including ICD interrogation. In the absence of VT recurrence at first follow-up, discontinuation of AAD was encouraged. Ventricular tachycardia recurrence was defined as occurrence of any sustained VT requiring ICD therapy, recorded within the ICD monitor zone lasting >30 s, or documented on 12-lead ECG, including VTs that occurred before discharge from the hospital. Mortality and its cause, LVAD implantation, and heart transplant during follow-up were recorded.

### Statistical analysis

Continuous variables are expressed as mean ± standard deviation or median with inter-quartile range (IQR) according to skewness and compared with Student’s *t*-test or the Mann–Whitney *U* test where appropriate. Categorical data are reported as numbers and percentages (%) and compared with the χ^2^ or the Fisher’s exact test. Time to occurrence of the primary outcome variable (sustained VT recurrence) was evaluated by survival analysis. Survival curves to assess event-free survival and comparison between subgroups (based on baseline and procedural variables) was estimated by the Kaplan–Meier method and compared by the log-rank test. Cox proportional hazards regression analysis was performed to identify predictors of VT recurrence. Independent predictors of VT recurrence were analysed with multivariable models using a backward stepwise selection approach. Variables for the model were selected based on univariable analysis (*P* < 0.10). At each step, the less significant variable was removed until all variables reached a *P* < 0.20. All tests were two-sided, and a *P*-value <0.05 was considered statistically significant. Analyses were performed using SPSS version 27.0 (IBM Corporation, Armonk, NY, USA).

## Results

### Patient characteristics

A total of 130 patients (mean age 69 ± 10 years, 87% men) were enrolled in six centres between March 2019 and December 2020. Forty patients (31%) were included in a high-volume referral centre where substrate modification based on EDP ablation for post-MI VT is the routinely performed ablation approach since 2013. The remaining 90 (69%) patients were included in five other centres without prior experience in EDP ablation. Left ventricular ejection fraction (LVEF) was 34% (IQR 25–43), 42% had an anterior MI and 28% had undergone acute reperfusion during the index MI. Prior to ablation, 28 (22%) patients had an electrical storm (≥3 ICD shocks/24 h) or incessant VT and 68 (52%) had ≥1 ICD shock. In 49%, amiodarone had failed to control VT. Baseline characteristics were comparable among patients ablated in centres with and without prior experience in EDP ablation. *Table 1* shows the patient baseline characteristics.

**Table 1:** Baseline patient characteristics

	All *n* = 130	Prior experience with EDP ablation *n* = 40	No prior experience with EDP ablation *n* = 90	*P*-value
Baseline characteristics				
Age, years	69 ± 10	70 ± 9	68 ± 10	0.326
Male, *n* (%)	113 (87)	32 (80)	81 (90)	0.158
Hypertension, *n* (%)	64 (52)	21 (53)	43 (48)	0.556
Diabetes, *n* (%)	34 (26)	10 (25)	24 (27)	1.000
Renal dysfunction (CrCl <60 mL/min), *n* (%)	38 (29)	12 (30)	26 (29)	1.000
Atrial fibrillation, *n* (%)	43 (33)	17 (43)	26 (29)	0.158
Anterior infarction, *n* (%)	54 (42)	13 (33)	41 (46)	0.129
CABG, *n* (%)	46 (35)	18 (45)	28 (31)	0.236
LVEF, % (IQR)	34 (25–43)	35 (30–45)	32 (25–41)	0.135
Arrhythmic presentation				
Electrical storm/incessant VT, *n* (%)	28 (22)	12 (30)	16 (18)	0.164
ICD shocks, *n* (%)	68 (52)	17 (43)	51 (57)	0.128
VT burden 6 months, *n* (%)	3 (1–10)	4 (1–20)	3 (2–9)	0.303
CL clinical VT, ms (IQR)	360 (310–440)	370 (300–430)	360 (310–455)	0.649
Prior anti-arrhythmic treatment				
Prior VT ablation, *n* (%)	22 (17)	6 (15)	16 (18)	0.803
Failed AADs, *n* (%)	81 (62)	28 (70)	53 (59)	0.315
Sotalol, *n* (%)	26 (20)	12 (30)	14 (16)	0.276
Amiodarone, *n* (%)	64 (49)	19 (48)	45 (50)	0.848
Amiodarone + Class I, *n* (%)	5 (4)	4 (10)	1 (1)	0.067
Heart failure treatment				
Beta-blockers, *n* (%)	100 (77)	28 (70)	72 (80)	0.179
ACEi or ARB or ARNI, *n* (%)	102 (78)	32 (80)	70 (78)	0.822
MRA, *n* (%)	52 (42)	13 (33)	39 (43)	0.250

Values are mean ± SD, median (IQR), or *n* (%).

AAD, anti-arrhythmic drug; ACEi, angiotensin-converting enzyme inhibitor; ARB, angiotensin receptor blocker; ARNI, angiotensin receptor neprilysin inhibitor; CABG, coronary artery bypass graft; CRT, cardiac resynchronization therapy; ICD, implantable cardioverter–defibrillator; LVEF, left ventricular ejection fraction; MRA, mineralocorticoid receptor antagonist; VT, ventricular tachycardia.

### Procedural data

Procedural data are summarized in *Table 2*. In total, 55 (42%) patients were on amiodaron during the procedure. Eighty-three (64%) underwent the procedure under general anaesthesia or deep sedation and one patient under mechanic circulatory support. In all but three patients, an endocardial only approach was performed (127, 98%). Image integration to facilitate the procedure was used in 46 (35%) patients and multipolar catheters (Pentaray, Biosense Webster, or HD Grid, Abbott) or a catheter with small-close-spaced electrodes (QDOT, Biosense Webster) were used for mapping in 70 (54%). A median of 1 (IQR 1–3) VT per patient was induced. Twelve (9%) patients were not inducible at baseline. Left ventricular substrate mapping following the pre-specified protocol including systematic analysis of EGMs with RV extra-stimulation was performed in 129 (99%) patients (one patient was repeatedly inducible for a VT while conducting the pacing protocol that could not be terminated by ablation). Evoked delayed potentials were identified in 122 (94%) patients and were the initial ablation target of ablation in 114 (88%). The remaining eight patients had repeatedly induction of tolerated sustained VT during the pacing protocol which was targeted by activation and entrainment mapping. After VT termination, further substrate mapping and ablation based on EDP was performed in all eight patients. After EDP ablation, 31 (24%) patients remained inducible for VT [median VTCL 310 ms (IQR 275–350)] but only in 8 (6%), VT was considered clinically relevant and targeted. The median procedural duration (from groin puncture to catheter removal) was 212 (IQR 179–262) min and the median RF time 23 (IQR 14–35) min. Re-mapping after ablation showed EDP elimination in 117 out of 122 (96%) patients in whom EDP ablation was attempted. In addition, PES was repeated in 115 (88%) patients, including stimulation with up to four extra-stimuli in 80/115 (70%). At the end of the procedure, any sustained VT remained inducible in 28 (22%) patients [median VTCL 300 ms (IQR 260–335)] but only in 4 (3%) patients the clinical VT was still inducible. Patients undergoing the procedure in the centre with prior experience in EDP ablation were inducible for a higher number of distinct VTs [median 2 (IQR 1–4) vs. 1 (IQR 1–2) VT per patient; *P* = 0.007] and for faster VTs [minimum VTCL 300 ms (IQR 270–340) vs. 345 (IQR 300–410), *P* = 0.002]. The median procedural and RF times were significantly shorter in the centre with prior experience in EPD ablation [median 175 (IQR 150–220) min procedural time and 13 (IQR 7–19) min RF time vs. 230 (195–280) and 32 (IQR 20–40) min, respectively, both *P* < 0.0001]. At the end of the procedure, a significantly higher number of patients in the experienced centre remained inducible for any VT (37% vs. 12%; *P* = 0.02). Procedural-related complications occurred in seven patients [5%; pericardial bleeding (*n* = 3), vascular access (*n* = 2), transient ischaemic attack (*n* = 1), and one procedure-related death (one patient with severely reduced LV function who underwent the ablation under ECMO support because of an electrical storm with fast non-tolerated VTs, died due to refractory cardiogenic shock days after the procedure].

**Table 2:** Procedural data

	All *n* = 130	Prior experience with EDP ablation *n* = 40	No prior experience with EDP ablation *n* = 90	*P*-value
Procedural data				
Number of induced VTs, *n* (IQR)	1 (1–3)	2 (1–4)	1 (1–2)	0.007
Maximum VTCL, ms (IQR)	370 (310–430)	350 (300–450)	390 (320–430)	0.586
Minimum VTCL, ms (IQR)	328 (290–400)	300 (270–340)	345 (300–410)	0.002
Non-inducible at baseline, *n* (%)	12 (9)	3 (8)	9 (10)	0.754
Amiodarone during ablation, *n* (%)	55 (42)	16 (40)	39 (43)	0.848
Mapping				
LV endocardium, *n* (%)	129 (99)	40 (100)	89 (99)	
RV endocardium, *n* (%)	26 (20)	5 (13)	21 (23)	
Epicardium, *n* (%)	3 (2)	1 (3)	2 (2)	
Target site identification				
Substrate mapping, *n* (%)	129 (99)	40 (100)	89 (99)	
Pacing protocol, *n* (%)	129 (99)	40 (100)	89 (99)	
EDP present, *n* (%)	122 (94)	37 (93)	85 (94)	
Activation/entrainment mapping, *n* (%)	50 (39)	14 (25)	36 (40)	0.590
Procedural time, min (IQR)	212 (179–262)	175 (150–220)	230 (195–280)	<0.0001
RF time, min (IQR)	23 (14–35)	13 (7–19)	32 (20–40)	<0.0001
Acute outcome				
EDP eliminated, *n* (%)	117/122 (90)	35 (88)	82 (91)	0.774
PES performed after RF, *n* (%)	115 (80)	35 (88)	80 (89)	0.638
Remaining VTs after RF				
Any VT, *n* (%)	26 (20)	15 (38)	11 (12)	0.002
Clinical VT, *n* (%)	4 (3)	3 (8)	1 (1)	
VTCL, ms (IQR)	300 (260–335)	300 (275–320)	310 (200–400)	0.838
PES not performed (reasons)				
Non-inducible at baseline, *n* (%)	12 (9)	3 (8)	9 (10)	
Only non-clinical VTs at baseline, *n* (%)	2 (2)	2 (5)	0 (0)	
Clinical VT not terminated by RF, *n* (%)	1 (1)	0 (0)	1 (1)	
Treatment at discharge				
ICD at discharge, *n* (%)	119 (92)	31 (78)	88 (98)	<0.0001
AADs at discharge, *n* (%)	69 (53)	23 (58)	46 (51)	0.570
Amiodarone, *n* (%)	54 (42)	16 (40)	38 (42)	0.849
Sotalol, *n* (%)	12 (9)	6 (15)	6 (7)	0.187
Class I, *n* (%)	3 (2)	1 (2)	2 (2)	1.000

Values are median (inter-quartile range) or *n* (%).

AAD, anti-arrhythmic drug; CL, cycle length; EDP, evoked delayed potentials; ICD, implantable cardioverter–defibrillator; LV, left ventricle; PES, programmed electrical stimulation; RF, radiofrequency; RV, right ventricle; VT, ventricular tachycardia.

### Post-procedural management

A total of 119 (92%) patients were discharged with an ICD, 31 (78%) patients treated in the experienced centre and 88 (98%) in the remaining centres. Eleven patients (9/11 in the experienced centre) were discharged without ICD after discussing the evidence supporting ICD implantation in each particular case. All patients discharged without ICD had presented with tolerated VT, 8/11 had an LVEF ≥ 40%, and 9/11 were rendered non-inducible after ablation. The majority of patients [69 (53%)] continued AAD after the procedure, with amiodarone prescribed for 54, sotalol for 12, and Class I AAD for 3; 11 patients stopped AAD immediately after an acutely successful ablation.

### Long-term outcome

Median follow-up after ablation was 14 (IQR 8–18) months [median 17 (IQR 9–23) months in the centre with prior experience in EDP ablation vs. 13 (IQR 8–17) months in the other centres].

**Figure 3:**
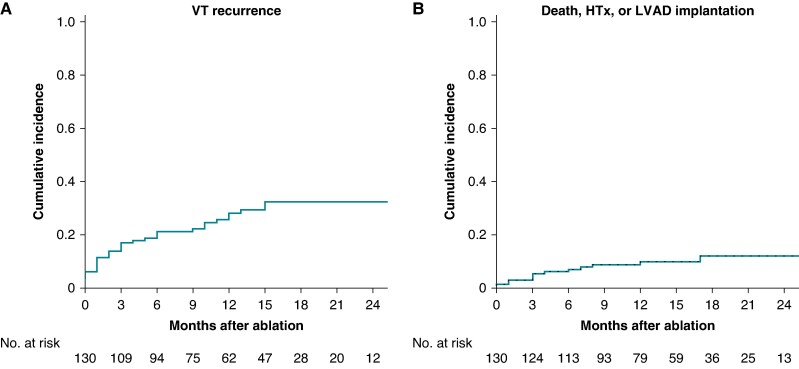
(*A*) Cumulative incidence of ventricular tachycardia recurrence after ablation. *(B*) Cumulative incidence of death, heart transplant, or LVAD implantation after ablation. HTx, heart transplantation; LVAD, left ventricular assist device; VT, ventricular tachycardia.

In total, 36 (28%) patients had VT recurrence after a median of 77 (IQR 17–195) days. Ventricular tachycardia-free survival was 78% (95% CI 71–85) at 6-month follow-up and 72% (95% CI 63–80) at 12-month follow-up (*Figure 3*). Out of 36 patients with VT recurrence, 10 had recurrent electrical storm (*n* = 3) or ICD shocks (*n* = 7), whereas the majority (*n* = 24) received antitachycardia pacing (ATP, *n* = 17) or had a tolerated VT under ICD detection (*n* = 7) only. Ventricular tachycardia burden decreased from a median of 3 (IQR 1–10) episodes of VT 6 months before ablation to a median of 0 (IQR 0–0) episodes of VT after ablation. Only one of the 11 patients discharged without an ICD had a recurrence of a tolerated VT at follow-up (an 86-year-old woman with an LVEF of 33% who was only inducible for non-clinical VTs during the procedure recurred with a slow tolerated VT). At last follow-up, 65 (50%) patients were still on AADs (51 on amiodarone).

A total of 13 patients died (*n* = 11) or received an LVAD (*n* = 2) during follow-up. No patient underwent heart transplantation. Mode of death was heart failure in five patients, a non-cardiac cause in three, arrhythmic/sudden cardiac death in two (both patients with ICD), and unknown in one patient.

### Long-term outcome according to centre experience with evoked delayed potential ablation

Ventricular tachycardia recurrence rate was not different among patients undergoing the procedure in centres with experience or without experience in EDP ablation [9/40 (23%) vs. 27/90 (30%); *P* = 0.406]. Six-month and 1-year VT-free survival rates were 82% (95% CI 70–95) and 76% (95% CI 62–90) in the centre with prior experience in EDP ablation vs. 77% (95% CI 68–86) and 70% (95% CI 59–81) in the centres without prior experience in EDP ablation, *P* = 0.319. Mortality rate was also not significantly different between centres [6/40 (15%) in the centre with experience vs. 7/90 (8%) in the centres without experience on EDP ablation, *P* = 0.218].

**Figure 4:**
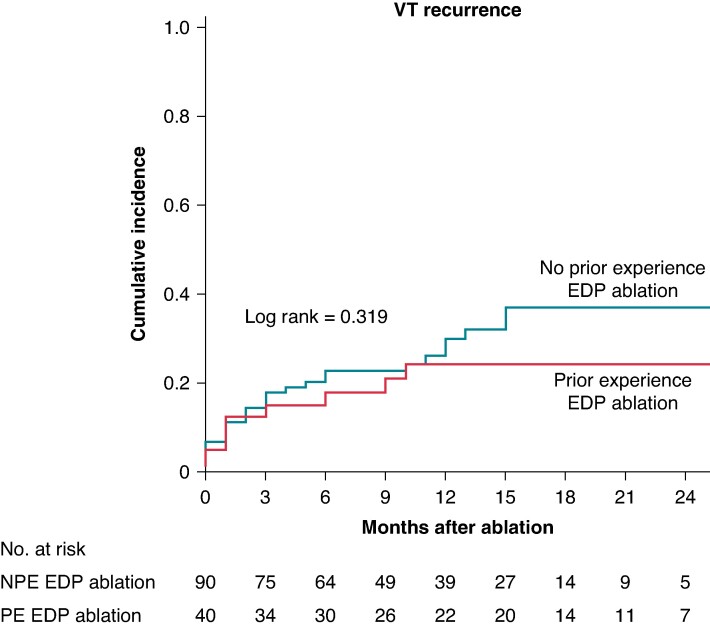
Cumulative incidence of ventricular tachycardia recurrence in centres with prior experience in EDP ablation vs. centres without prior experience in EDP ablation. EDP, evoked delayed potential; VT, ventricular tachycardia.

**Figure 5:**
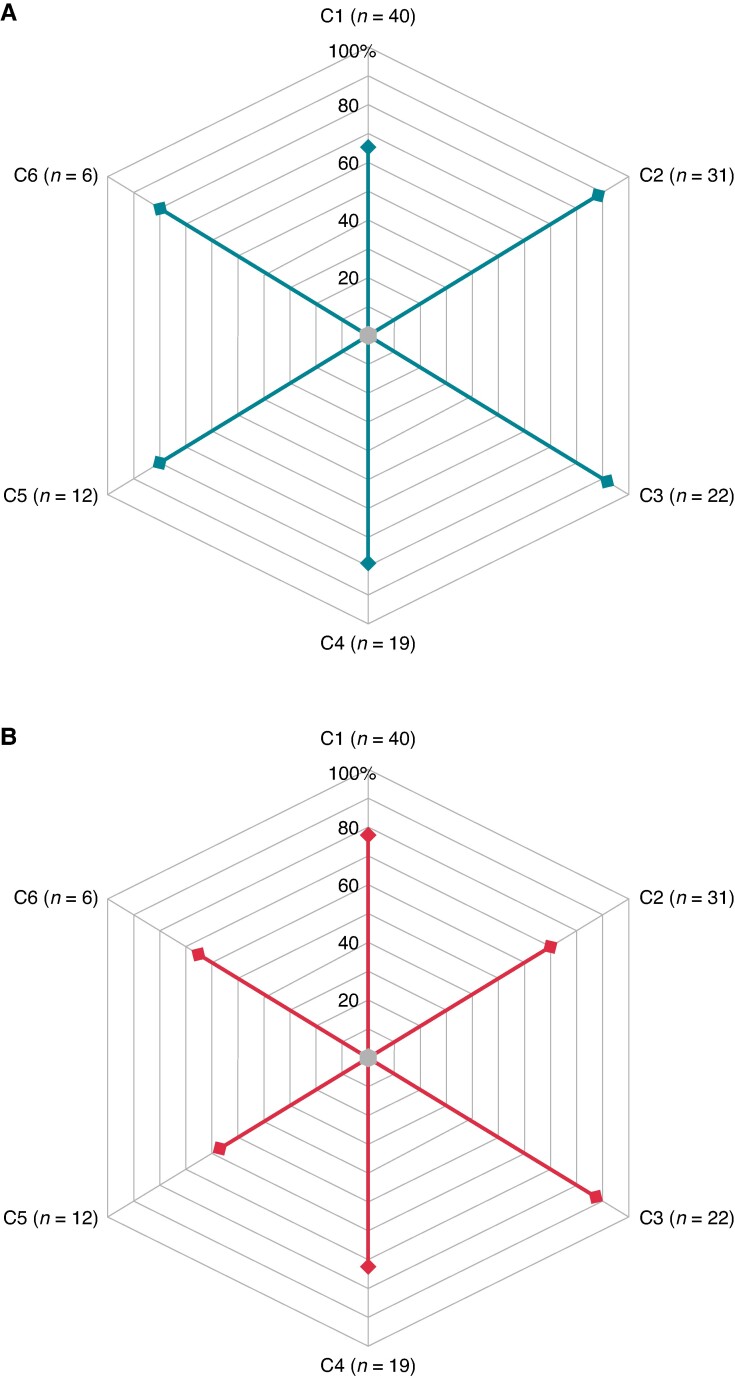
Acute and long-term ablation outcomes per centre. (*A*) Non-inducibility after ablation and (*B*) VT recurrence.

In univariable Cox regression analysis, a lower LVEF was the only factor associated with VT recurrence [hazard ratio (HR) 1.05 per 1% decrease in LVEF (95% CI 1.02–1.08), *P* = 0.03] and the only one remaining significantly associated with VT recurrence on multivariable analysis [HR 1.05 per 1% decrease in LVEF (95% CI 1.05–1.09), *P* = 0.04]. Of importance, the cumulative incidence of VT recurrence did not differ among patients undergoing the ablation in centres with or without prior experience in EDP ablation [HR experienced centre 0.687 (95% CI 0.322–1.462), *P* = 0.330] (*Figure 4*). Other factors, such us presentation with electrical storm/incessant VT, use of amiodarone, number of induced VTs during the procedure, or VT inducibility after ablation, were not associated with VT recurrence (*Table 3*). The acute outcome (VT non-inducibility) and 1-year VT recurrence per centre are depicted in *Figure 5*.

**Table 3:** Cox regression analysis for predictors of the combined endpoint of VT recurrence

	Univariable		Multivariable	
	Hazard ratio (95% CI)	*P*-value	Hazard ratio (95% CI)	*P*-value
Centre with prior experience in EDP ablation	0.687 (0.322–1.462)	0.330		
LVEF (per 1% decrease)	1.05 (1.02–1.08)	0.03	1.05 (1.05–1.09)	0.04
Electrical storm/incessant VT	1.049 (0.478–2.302)	0.905		
Amiodarone at admission	0.880 (0.456–1.701)	0.704		
Number of induced VTs (per additional VT induced)	1.146 (0.911–1.442)	0.243		
Inducible after ablation	1.286 (0.604–2.736)	0.514		—

EDP, evoked delayed potentials; LVEF, left ventricular ejection fraction; VT, ventricular tachycardia

An older age [HR 1.09 per 1-year increase (95% CI 1.01–1.18), *P* = 0.028] and a lower LVEF were associated with a higher probability of dying or receiving an LVAD during follow-up [HR 1.05 per 1% decrease in LVEF (95% CI 1.01–1.10), *P* = 0.044]. Both factors remained associated with a higher probability of death/LVAD implantation after multivariable analysis. The cumulative incidence of death/LVAD implantation was not different in centres with and without prior experience on EDP ablation.

## Discussion

### Main findings

In this multicentre international prospective study including a large cohort of contemporary patients with post-MI VT, substrate ablation based on the identification and elimination of *EDPs* was associated with a low VT recurrence rate at 1-year follow-up of 28%. Of importance, survival free from VT was comparable for patients undergoing the procedure in a high-volume referral centre for VT ablation where EDP-based ablation had consistently been performed for 6 years and on more than 100 post-MI patients and for patients who were treated in five centres without prior experience with this specific ablation approach. These data suggest that identifying and targeting EDP by ablation can be easily generalized, with consistent and favourable clinical outcomes. A lower LVEF was the only predictor of VT recurrence on multivariable analysis after correction for clinical and procedural factors previously reported to be associated with post-MI VT ablation outcome, including inducibility after ablation.

### Substrate ablation for post-myocardial infarction ventricular tachycardia

After MI, only one-third of the patients referred for VT ablation are exclusively inducible for tolerated, mappable VTs allowing for identification of critical VT sites by detailed activation and entrainment mapping.^13^ However, restricting ablation solely to clinical and stable induced VTs has been associated with a higher VT recurrence rate compared with a substrate-based ablation approach.^1^ Consequently, substrate mapping and ablation is currently performed in the majority of post-MI VT ablation procedures, alone or, when VT is stable, in combination with VT mapping.^2^

Multiple approaches for substrate modification have been proposed, with most focusing on the detection of EGMs exhibiting characteristics indicative for abnormal conduction, which is a prerequisite for reentry.^4,5,7^ However, most of the data on the efficacy of these approaches come from retrospective non-controlled studies conducted in high-volume referral centres or that include mixed populations of patients with ischaemic and non-ischaemic cardiomyopathies, who may have different underlying VT substrates.^5,7–9^ Of importance, proposed ablation targets are variable [e.g. late potentials with onset after QRS separated by an isoelectric segment >20 ms, fragmented EGMs with > 4 positive sharp deflections and an amplitude/duration ratio ≤ 0.005, or any abnormal EGM with a duration >70 ms and/or a ratio amplitude/duration >0.046] and often have a broad definition, which can lead to individual interpretation, potentially making the outcome of the procedure dependent on the operator’s experience.^5,6^ For example, the definition of local abnormal ventricular activities includes a wide spectrum of EGMs with low- or high-voltage sharp components, occurring at any time before, during, or after the far-field EGM. These EGMs can be found in large border zone areas.^5^ Studies on the reproducibility of outcomes associated with ablation approaches targeting EGMs considered to be abnormal or related to VT are scarce, especially evaluating recently implemented mapping and ablation approaches.^8,9^

### Evoked delayed potential-based substrate ablation for post-myocardial infarction ventricular tachycardia: procedural findings and acute outcome

In a prior single-centre retrospective study including 60 patients, we could demonstrate that substrate modification based on EDP ablation was feasible, safe, and highly effective for the treatment of post-MI VT.^10^ Evoked delayed potentials are distinctly characterized as near-field low-voltage EGMs that exhibit delay of >10 ms or block when subjected to a single short-coupled RV extra-stimulus, indicating regions with functional slow conduction properties, which are more likely to be associated with VT. Because of a clear and measurable definition, the centres participating in the current registry who had no prior experience in this ablation approach were able to start performing the procedures based on EDP identification after a short training. Of note, the pre-specified RV pacing protocol was successfully completed in 99% of the patients, resulting in identification of EDPs in 94% of cases. This significantly surpasses the identification rate of other proposed specific targets such as late potentials which have been found in only two-thirds of patients with post-MI VT.^14^ Initial ablation was based on EDP in 88% of the patients, and after EDP ablation, only 24% remained inducible for any (mainly non-clinical) VT. The high adherence to the protocol is encouraging, indicating that it is operable and practical. Ablation of induced VTs before finalizing EDP ablation was only required in eight patients (6%).

Patients in the experienced centre were inducible for a higher number of VTs throughout the procedure (median 2 vs. 1) and also remained inducible more often after ablation. This may be partly explained by variation in the application of the induction protocol (e.g. number and selection of LV sites, 350 ms, and 4 extras at all sites) at the end of the procedure, since it did not translate into poorer long-term outcomes. The median procedural duration was 212 min, which is similar or shorter than reported by other studies evaluating different substrate modification approaches.^4–7,15^ In the experienced centre, the procedural duration was significantly shorter, with a median time of 175 min, indicating that with increasing experience, the procedure might be completed within 3 h. This contradicts the concerns that conducting such a pacing protocol might unacceptably extend procedural duration. The median RF time was also short with a median of 23 min and significantly shorter in the centre with prior experience on EDP ablation (median 13 min). This observation underscores the specificity of EDPs as a target and highlights that a focused approach on EDP identification may reduce excessive ablation of myocardium potentially contributing to cardiac function. Of importance, the complication rate was low (5%), supporting the safety of the approach. Notably, 11 patients were discharged without an ICD, with the majority (9/11) from the experienced centre. This has recently been shown to be a safe strategy in highly selected post-MI patients with LVEF > 35% presenting with tolerated VT, provided that they are rendered non-inducible for any VT after ablation.^16^

### Evoked delayed potential-based substrate ablation for post-myocardial infarction ventricular tachycardia: long-term outcome and predictors of ventricular tachycardia recurrence

Ventricular tachycardia-free survival rates after post-MI VT ablation have been reported to range between 53 and 75% during a median follow-up of 6–24 months.^9,13,14,17,18^ Consistent with these findings, VT-free survival for the entire cohort in our study was 78 and 72% at 6-month and 1-year follow-up, respectively. Importantly, VT recurrence rates were not significantly different between the centre with extensive experience in EDP-based ablation and the centres without prior experience with this technique, supporting the reproducibility of outcomes using this ablation approach. Similar to previous reports, patients with a lower LVEF had higher rates of VT recurrence and death.^10,19,20^ On the contrary, unlike prior analyses, a higher number of inducible VTs during the procedure and inducibility after ablation were not associated with a poorer long-term outcome. This could be partly explained by the low median number of inducible VTs (only one per patient) compared with previous studies, which might be attributed to a modern approach to VT ablation that focuses more on substrate identification during stable rhythm than VT mapping.^13,20^ Furthermore, the majority of patients who remained inducible after ablation were only found to be inducible for fast VTs, the clinical significant of which remains a subject of ongoing debate.^11^

### Study limitations

This study has the limitations of an observational study. However, all the data were acquired prospectively and a common protocol of patient inclusion, mapping, ablation, and follow-up was applied in all the participating centres. Interruption of AADs at first follow-up was recommended in the absence of VT recurrence but ultimately left at the discretion of the treating physician. This might have influenced outcomes in some patients. Randomized studies to directly compare EDP ablation with other substrate modification approaches in post-MI patients are warranted.

## Conclusions

In this large multicentre international prospective cohort of contemporary post-MI VT patients, substrate modification based on EDP ablation resulted in favourable long-term outcomes. Of importance, VT recurrence rates did not differ among centres with and without prior experience in this ablation approach, indicating that this approach can be easily reproduced by operators previously not familiar with the technique.

## Supplementary Material

euaf003_Supplementary_Data

## Data Availability

The data supporting the findings of this are available upon reasonable request to the corresponding author.
