# Supplementary figures and images for: Evoked delayed potential ablation for post-myocardial infarction ventricular tachycardia: results from a large prospective multicentre study
Source: Europace. 2025 Feb 24;27(2):euaf003. doi: 10.1093/europace/euaf003 (PMC11848844; doi:10.1093/europace/euaf003)

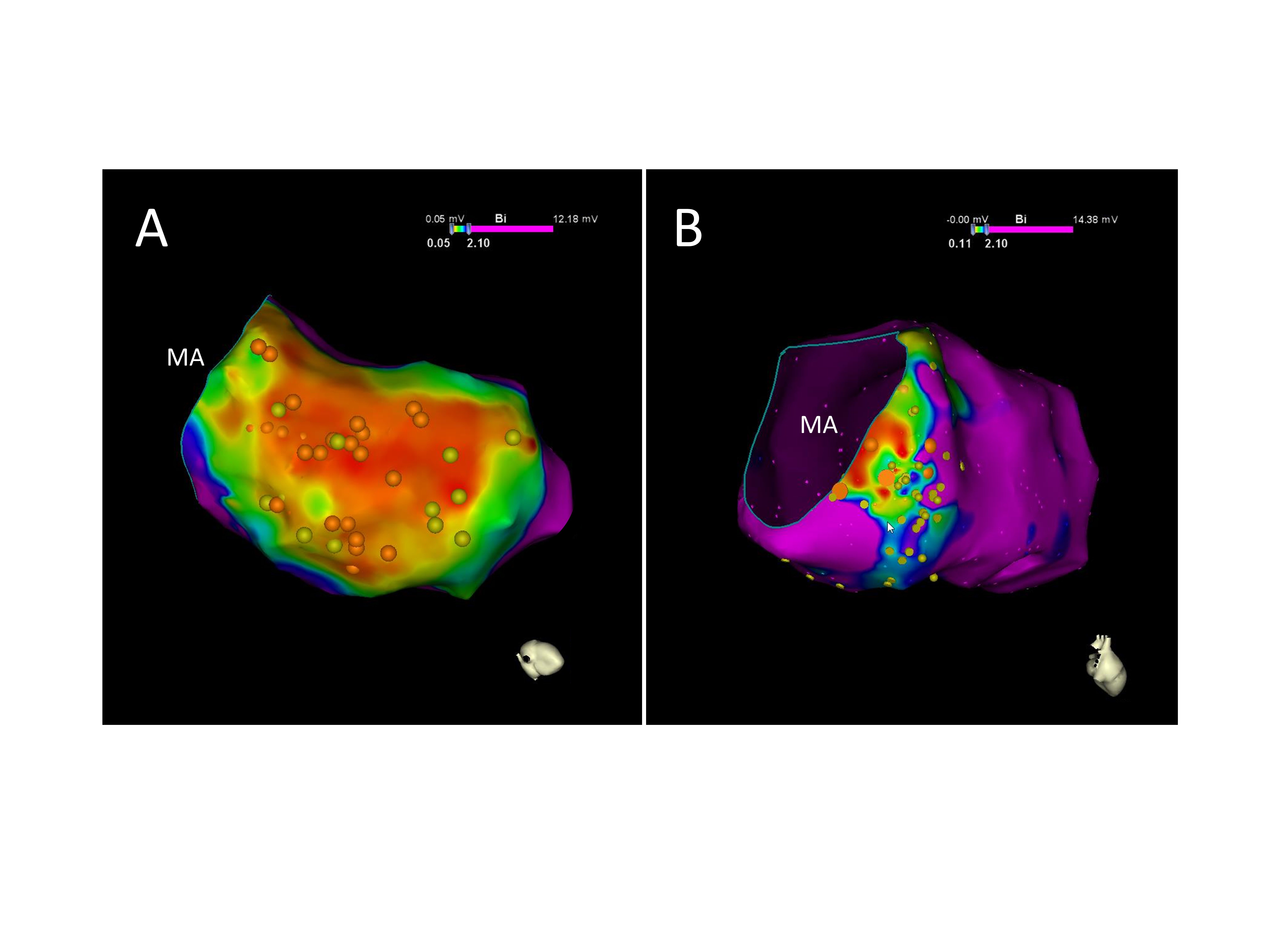

Supplement: euaf003_Supplementary_Data [file euaf003_supplementary_data.jpeg]
